# From Restricted Resources to Ethical Burden—Former Home Care Workers’ Reasons for Leaving Their Jobs

**DOI:** 10.1177/07334648241231404

**Published:** 2024-02-14

**Authors:** Marjo Ring, Hanna Ristolainen, Elisa Tiilikainen

**Affiliations:** 1 Department of Social Sciences, Faculty of Social Sciences and Business Studies, 163043 University of Eastern Finland, Kuopio, Finland

**Keywords:** home care, older adults, shortage of nursing, care work, new public management

## Abstract

The study examines former home care workers’ reasons for leaving their jobs from the perspective of reforms in public services and eldercare policies impacted by New Public Management (NPM) in Finland. Written narratives from former home care workers (*n* = 39) were collected online and analyzed using thematic content analysis. Former home care workers’ reasons for leaving their jobs were connected to four interconnecting themes: *mismatch between needs and resources*, *measurement-driven practices*, *unbalancing work*–*life*, and *ethical burden*. These reasons reflected critical changes in the organization of care work and the work environment in older adults’ home care. Contradictions between needs, resources, and values lead to ethical dilemmas and push away from the workforce in eldercare. To improve care workers’ willingness to remain in the eldercare sector, changes are needed in the resourcing and organization of home care, including managerial support in everyday care work.

**What this paper adds**

• Increases understanding of home care workers’ reasons to leave their jobs in public eldercare services.
• Reflects the impacts of New Public Management reforms on the lived experiences of home care workers working with older adults.
• Elucidates the structural circumstances challenging the quality of work–life and care work in public eldercare services.

**Applications of study findings**

• Home care workers’ intentions to leave their jobs need to be addressed at a structural level to improve the quality of care work in eldercare services.
• More emphasis is needed on home care workers’ understanding of good care when planning and implementing reforms in the eldercare sector.
• Open discussions on the values of care work are needed between professionals and policy makers when aiming to maintain workforce in the eldercare sector.

## Introduction

In many countries, long-term care services for older adults are suffering from a shortage of workers, such as nurses and other professionals in the care sector (OECD, 2022). Long-term care workers are dissatisfied with their salaries, working conditions, career prospects, and the increasing physical and mental stresses of job (Van Aerschot et al., 2022). This has led to difficulties keeping professionals in their jobs and recruiting new workforce, making it challenging, to provide the necessary services needed to meet the demands of aging populations (Rostgaard et al., 2022). In this article, we examine Finnish home care workers’ reasons for leaving their jobs from the perspective of reforms in public services and eldercare policies impacted by New Public Management (NPM).

The basic idea of NPM has been to transform public sector organizations into market-oriented and business-like service providers (Diefenbach, 2009). The NPM reform—rooted in 1980s—was fundamentally a response to increased dissatisfaction towards governments’ abilities to maintain economic and social development (Keating, 2001). New Public Management integrates goals, means, and rules that contain value statements of how public organizations should be designed, organized, and managed in order to rationalize and optimize the use of resources and to achieve higher efficiency and effectiveness (Diefenbach, 2009; Kamp, 2012; Lane, 2000; Mauri 2012; Simonet, 2015). This is understood to happen through strategic planning and management, emphasis on standardized work methods, customer orientation, and performance measurement (Diefenbach, 2009).

Nordic countries—Denmark, Norway, Sweden, and Finland—have all applied versions of NPM as a solution to what were perceived as shortcomings of the state (Dahl & Rasmussen, 2012). In Finland, the NMP reform has had a significant role in reforming public administration since late 1980s and early 2000s (Lähdesmäki, 2003). Moreover, Finland has been referred to as a model country of NPM (Herranen, 2015) and the importance of public administration reforms seems to be stronger here than in many other countries (Virtanen, 2016). This has been evident also in the eldercare sector, which has been impacted by a wide range of marketization during the past decades (Anttonen & Häikiö, 2011; Karsio & Anttonen, 2013).

At the beginning of the 1990s, economic recession led to severe expenditure cuts in public services, creating a suitable environment for administrative reforms. The radical decentralization of administration made local authorities significantly more independent on central government, increasing their financial pressure and leading to drastic cuts in public services, including older adults’ long-term care. (Kröger & Leinonen, 2012.) The perspectives and values of private business mechanisms and principles were considered the primary means for the government to maintain the public sector and enable public organizations to better respond to citizens’ needs and ensure more efficient provision of services (Diefenbach, 2009; Rasmussen, 2012). The ideological background of these changes can be found in NPM (Lane, 2000).

NPM is a government level reform, but its effects are reflected at the practical level of organizations (Lane, 2000). The reform has been criticized for its lack of suitability with the practical level of service provision and its implementation. There have been significant undesirable side effects and misfits between policy announcements and implementation (Simonet, 2015). NPM’s strategic orientations have been specifically criticized for being based on too artificially and narrowly designed concepts of measurement and accountability (Diefenbach, 2009). Through these orientations, NPM has been seen to challenge the foundations of care work (Dahl & Rasmussen, 2012). Within the context of eldercare services, the main component of NPM is the standardization of work in order to allow centralized control (Kamp, 2012, also Tufte & Dahl, 2016). Managers construct their identities by embracing the logic of NPM, as care workers build their identities based on professional practices. When these are in conflict, the meaningfulness of work is challenged, and care workers experience loss of autonomy (Keisu et al., 2016.) However, care workers are not passive recipients, as they have also opposed to the NPM discourse and raised concern about the changes implemented (Dahl, 2009).

Previous research examining Finnish eldercare professionals’ intentions to leave their jobs have found that they are impacted by a lack of opportunities to influence the daily planning of work, feelings of inadequateness due to care recipients not receiving enough help, and care workers not having time to carry out the tasks required. Also, immediate support from supervisors was perceived as insufficient and care workers experienced psychophysical burden. Intentions to leave the job were also related to worries about income (Van Aerschot et al., 2022). According to studies from other countries, nurses' turnover has been found to be caused by lack of social support, work overload, low-level job satisfaction, and poor working conditions. In addition, among older nurses, the required use of new technologies was one reason given for leaving the profession (Tamata & Mohammadnezhad, 2023). Nurses' common reasons for intention to leave were working conditions (e.g., time pressure and quality of care) and family reasons (e.g., caring for relatives) (Estryn-Behar, Van der Heijden, & Hasselhorn, 2010). In the United States, up to 20% of nurses working in hospitals were found to be intending to leave their position (Koehler 2022). In the Nordic countries, 41% of eldercare workers have considered leaving their current job (Van Aerschot et al., 2022).

In addition to NPM reforms, Finnish eldercare services have undergone a process of de-institutionalization aimed at supporting older adults’ possibilities to live in their own homes (Kröger, Puthenparambil, & Van Aerschot, 2019; Pulkki et al., 2017). However, compared to other European and Nordic countries, resources for older adults’ home care have decreased alongside other forms of long-term care (Kröger & Leinonen, 2012; Rostgaard et al., 2022). Currently, 14.8% of Finnish older adults aged 75 and over are clients of regular home care (Sotkanet, 2023), which is mainly provided by the public sector. Over half (59%) of home care clients have a frequent need for assistance in their daily activities, and almost one fifth (18%) are visited by home care workers three or more times a day (Saukkonen et al., 2021). At the same time, formal home care has been shifted from a holistic social care service (including housekeeping, for example) to more medically focused home nursing (Kröger & Leinonen, 2012).

Organizational reforms and changes in the working environment have a significant impact on the everyday work done by professionals in eldercare (Strandås et al., 2019) and may lead to lack of motivation to stay in the job (Van Aerschot et al., 2022). This article adds to existing knowledge by examining former home care workers’ reasons for leaving their jobs in Finnish eldercare services from the perspective of New Public Management. The research questions were:

• What have been the care workers reasons for leaving their positions in older adults’ home care?
• How do care workers’ reasons for leaving their positions reflect the New Public Management reform?

We addressed these questions by examining written narratives of former care workers who have previously been working in Finnish formal home care services.

## Methods

The data consisted of written narratives (23 pages) from 39 former home care workers who had left their job in Finnish eldercare services. Background information of the study participants is described in Table 1. A quarter of respondents were registered nurses (bachelor degree), and the rest were practical nurses (Vocational Qualification, two-year education). The data was collected between September and November 2021 using the “Penna” textual data collection tool maintained by the Finnish Social Science Data Archive (FSD). The invitation to participate was shared on social media via Facebook, Twitter, and LinkedIn and was available to share in other platforms and networks. The respondents were asked to describe their thoughts on why they have left their nursing position. The request was intentionally very open so that respondents could freely bring up issues most relevant to them. Altogether the original data set consisted of 295 narratives from nurses who have left the healthcare sector. For this study only narratives from former home care workers were used.

**Table 1.** Age, Education, and Working Years of Respondents (*n* = 39).

	n
Age	
20–24	2
25–34	15
35–44	15
45–54	7
Education	
Practical nurse	29
Registered nurse	10
Working years	
1–4	10
5–9	11
10–14	13
14–20	5

The data was analyzed using thematic analysis to examine the experiences and descriptions of participants’ work–life before leaving their job in home care. Our analysis was data-driven as the coding process did not fit a pre-existing coding frame or the researchers’ analytic preconceptions (Braun & Clarke, 2006). First the data was read by all authors to gain an overall understanding of the participants’ experiences related to their work in home care. Second, each author started analyzing the data separately by selecting, coding, and grouping data excerpts relevant to our research question. These preliminary findings included themes, such as “changes in work conditions,” “ethical and emotional burden,” “change in client work,” “ethical dilemmas,” “feelings of inadequacy,” and “functional deterioration of clients.” In the third phase of the analysis, all findings were jointly discussed and reflected on by all three authors several times. At this point, preliminary themes were merged into four key themes that were found to illustrate the most dominant findings regarding the participants’ reasons for leaving their jobs: “mismatch between needs and resources,” “measurement-driven practices,” “unbalanced work–life,” and “ethical burden.” In the final phase, these key themes were interpreted from the perspective of the NPM reform.

## Findings

The results showed that the former home care workers’ reasons for leaving their jobs were connected to four interconnecting themes: mismatch between needs and resources, measurement-driven practices, unbalanced work–life, and ethical burden. Within the main themes we identified key NPM perspectives, which will be further reflected on below for each theme.

### Mismatch Between Needs and Resources

Care workers indicated that during their career home care clients’ health and functional abilities had declined in general. At the same time, resources had become limited and formal home care had shifted from holistic care practice to medically focused care. The home was seen as a “storage place” for clients where only mandatory care needs were met and all provision for other care needs had become stripped away. The task of the public sector was to provide support only for older adult’s daily living. Other services for maintaining everyday life (e.g., cleaning) had to be obtained by the clients themselves from the private sector.

I used to work with older adults in home care. During that time, home care changed a lot. Things like cleaning stopped and the visits became home nursing. The older adults had to buy all other necessary services from private service providers. They were left in home care in increasingly worse health, and institutional care was run down. People with memory disorders were either locked in their homes or reminded to use the emergency bracelet. The responsibility for these fell on the most recent nurse. Why is that since they wouldn’t even have wanted to leave them there. You were always afraid of what you might find in the morning shift or that you perhaps wouldn’t find anything at all (age 35−44, work experience >10 years).

In recent decades, places of round-the-clock-care have been drastically reduced, which was seen as a key barrier to older adult’s access to assisted living and other forms of round-the-clock care. This had led to a situation where older adults with multimorbidity and very limited functional abilities were forced to live at home “for too long.” For the workers, this was seen as a way to save costs, rather than supporting older adult’s right to live in their own home. This burdened the care workers' ability to work, as they felt that they are the ones responsible for providing this support. Care workers expressed being worried and afraid about clients’ ability to cope alone at home.

While the condition of the clients is deteriorating, people are treated only to be discharged, after which they die at home. End-of-life care, people who do not want to stay at home anymore, people who are sent to and from between home and hospital when their place isn’t in either but no other solution exists. There are no placements available and today’s trend is all about making savings (age 25–34, work experience 2–3 years).

According to the care workers, customer orientation—emphasized in NPM—was not realized in home care or it required the clients' own financial possibilities to participate in the procurement and payment of services. Customer-oriented care provision appeared only as a response to mandatory healthcare needs. Instead of comprehensively considering and addressing the needs of older adult, client orientation was narrow and limited to the conditions offered by the organization. Moreover, private services were not available to everyone due to limited financial resources and regional variation in the services offered, and therefore the possibility to choose between services was realized only for some home care clients.

### Measurement-Driven Practices

Care work was tied to accounting performances and auditing clients’ outcomes and work efficiency following the aims of NPM. To evaluate whether the client’s needs were addressed, several standardized measurements were used to measure the older adult’s functional abilities, which had changed care work into a technical performance. Care workers felt that their autonomy had decreased because their opportunities to influence the content of their work were limited. Demands for measurement and the use of specific technologies determined how care practices were carried out. Moreover, the time used for measuring and reporting measurement outcomes was taken away from actual care work. Care workers pointed out that working with clients’ diverse needs and changing situations was poorly suited to meeting rigid standards and tight time frames but felt that their voices were not heard.

The older adults living at home were increasingly multimorbid. Record keeping became even more important with the adoption of mobile devices. Clients had to be treated based on all kinds of metrics, the amount of paperwork increased a lot and replaced actual care, so to speak. Little by little, the work began to feel like a burden, it wore me down (age 35–44, work experience >10 years).

Work performances were monitored and controlled by different enterprise resource planning systems, which are widely used in home care in Finland. The purpose of these software solution systems is to organize work efficiently and to produce information for managers. However, these systems appear inoperative and inadequate from the workers’ perspective. When work is planned based only on the time limits of standard visits, allocated times for care work are too optimistic, making daily work and home care visits continually rushed. Moreover, care workers were unable to carry out individual care because the number of clients was too large. Participants described not being able to do their work as well as they would like and the clients deserve. At the same time, optimized time limits burdened the care workers by preventing them from taking breaks during their shifts.

According to the care workers’ descriptions, the amount of work tasks in older adult’s home care had increased significantly, making it impossible to complete all tasks within the times specified by resource planning system. As a NPM-based tool, the aim of enterprise resource planning systems is to optimize the use of working time and the timeliness of reporting, but, in practice, the systems did not sufficiently take into account the changing needs of clients and the realities of the work. Home care practices were guided by performance and measurement instead of considering the practicalities of care work processes.

At first, I liked my job when home care was organised by the municipality. The working days were reasonable, concentrated on the morning shift, the clients were cheerful. With the change to the joint municipal authority, everything shifted. The number of beds in hospitals was reduced, nursing homes were no longer accessible, but there seems to always be room in home care. As a result of the joint municipal authorities, I started doing 25–30 client visits in one shift, while previously there had been about 10 visits a day. In addition to the 30 client visits, the drive could be more than 100–150 km. All this had to be carried out during the working day. The mobile devices were already sending warnings about overly long days, even though the transition times hadn’t even been counted towards the day plan. Needless to say, I could only spend a couple of minutes per client […] Familiarizing yourself with the situation and practices of several hundred clients in their homes is impossible, you’re lucky if you’re able to find your way to the destination while the mobile device is telling you you’re already several hours behind schedule. Everyone was annoyed when the nurse was too late. The clients’ need for care grew exponentially when they no longer felt safe (in connection with the shift to the joint municipal authority) since they didn’t get enough time from the nurse during the morning visit (age 25−34, work experience >10 years).

Optimizing working times to only accommodate mandatory tasks does not seem to support customer orientation in care work. According to the participants, if clients’ needs were not met adequately, the need for care and support increased. Mobile devices and measurement are functional in planning work and monitoring its results, but, based on the data, enterprise resource planning systems do not fit the practices of complex and multidimensional care work. Care workers had little opportunities to influence planning their work or work shifts.

### Unbalanced Work–Life

According to the care workers, work–life was seen as unbalanced due to the distance of managers from everyday care work, demands for flexibility, and the negative effects of work on personal life. Additionally, there was an on-going shortage of care workers, which increased the amount of work tasks for employed workers. Care workers felt obliged to work in a hurry and do overtime. Operational unit sizes had increased due to enterprise resource planning systems distancing managers from employees. At the same time, management became more professional, separating managers from the practical work. Employees felt managers no longer understood the real challenges and demands they encountered in their work. Participants described managers pressuring them to work overtime due to lack of staff.

I kept getting calls asking me to work overtime, although the working days were already filled with too much work. I coped for half a year and burned out. I was on sick leave for a long time and I had lost all my enthusiasm for work. I felt like I was giving it everything I had, but it wasn’t enough. In the end, I decided I couldn’t continue like this and changed profession. For me, it’s been the best decision. I liked the work, but I couldn’t cope with the working conditions (age 20−24, work experience 2 years).

Employees felt obliged to be flexible—coming to work on short notice and giving up their free time—because they did not want to leave clients without care. They felt they were never able to recover from work as they had to be continually ready to cover for absent colleagues. Despite these demands for flexibility, care workers felt that employers and managers were not flexible towards them. Flexibility appeared to be one-sided. Ignoring care workers’ rights, having too little staff and too tight timetables for carrying out the work caused mistrust, rigidity and discord among the care workers. Participants felt their arguments and wishes were not listened to by managers. According to the data, positive incentives were not used as a tool for personnel management—as present in the NPM ideology. Management was rather seen as authoritarian demands for work performances and no attention was paid to the wellbeing of the employees. The duty of the care workers appeared to be to fulfill the tasks assigned to them under the control of the managers.

Some of my co-workers resigned, and no replacements were found and no new employees were recruited, which meant that someone often had to do a “long” shift. The atmosphere at the workplace began to grow tense, since most people were tired of the situation (age 25–34, work experience 2 years).

Care workers indicated that they did not get support from managers to correct deficiencies in their work, which increased the sense of being unable to provide proper care. The work of older adults’ home care is, by nature, independent and done alone. The optimization of working time has limited opportunities for joint meetings where they could share their experiences and the challenges of their daily work with other care workers. Care workers felt more and more forced to carry the burden of their work alone. Feeling burdened and being on constant standby also had negative impacts on family life, as any time off work was spent attempting to recuperate from the heavily demanding and hectic work.

For almost 2 years there haven’t been any meetings at work, you make all decisions alone, you bear the responsibility alone, you carry the ethical burden inside you and can’t unload it anywhere. When you get the odd day off, you’re tired, you lose all your energy just thinking about your shifts and when you’ll be able to take care of this and that and what you can just leave be. At home, you feel completely numb, you don’t want to talk to anyone, you don’t want to socialize or make any plans. You are angry, apathetic and just keep staring straight ahead, paralyzed (Age 25–34, work experience 2–3 years).

### Ethical Burden

Former care workers experienced anxiety and ethical burden because they were not able to do their work the way they wanted. The professional demands placed on workers and the aim of carrying out needs-based care work within limited time resources increased the care worker’s ethical burden, which was seen as a crucial reason for leaving the workforce.

Ethical dilemmas were faced by care workers when encountering individuals who had to manage at home without adequate support. Due to limited time, care workers could not stay to provide clients with the further help they needed. Ethical dilemmas were also seen in situations where an older adult no longer wanted to live at home, but round-the-clock care was not offered due to limited possibilities for placement in assisted living. Concern about older clients’ ability to manage daily life and the awareness that it should be the job of workers in the care sector to provide help where needed, caused ethical conflict for many care workers.

I thought the work would be nice, people-oriented and that I would be able to help others. At first everything was fine, I enjoyed the work, but with time the burden started to weigh me down. I could no longer perform as well as I would’ve liked, the working days weren’t enough to carry out the work and finally I had a burn-out (age 25−34, work experience 5 years).

Participants described being in constant ethical conflict in their daily work and feeling inadequate as they could not respond to older adult’s care needs. Moreover, they felt they were the ones who had to face the clients’ disappointment. How care workers would like to do their work and how they were required to do it appeared at odds. Workers’ expectations of a client-centered way of working according to their own values were not fulfilled. Having to work against their own professional competence and ethics also made care workers disappointed in themselves, which was corrosive to their professional identity. Moreover, the fight to improve client wellbeing and health was seen as exhausting, as there was no prospect of better times coming. For most participants, ethical and moral exhaustion led to leaving their job and changing profession.

Many times I left work crying because I wasn’t allowed to do my work properly. The last client in the morning often received their morning medication at 12:30 and was already eating the food provided by the catering service since they had missed breakfast. People with memory disorders can no longer make sure that they eat properly themselves. I always reported any problems to my managers, for years, and nothing changed (age 45−54, work experience >10 years).

## Discussion

In this study, we have examined home care workers’ reasons for leaving the job and how these reasons reflect structural changes in the services of older adults from the perspective of New Public Management. Our results showed that care workers have left their job in home care due to four main reasons: mismatch between needs and resources, measurement-driven practices, unbalanced work–life, and ethical burden. The key findings show that there are several contradictions between the principles of NPM and care work, which for many care workers may lead to ethical dilemmas and push them away from the eldercare workforce. Our findings are in line with previous studies that have found care workers struggling with excessive workload (Koehler & Olds, 2022) and facing challenges in responding to clients’ needs (Paavolainen et al., 2023; Selader et al., 2022). However, research focusing on former home care workers’ reasons for leaving the workforce has been scarce and, to our knowledge, non-existent from the perspective of New Public Management.

The basic element of NPM is efficient provision of services, which has manifested as an increased demand for measuring the performance of care workers. Time intended for the client has shifted to reporting to the organization, which is in conflict with care work itself: workers are unable to work according to their professional values by meeting older clients as individuals and recognizing their individual care needs. At the same time current aging policies emphasize older adult’s ability to live at home as long as possible, and older home care clients have more functional disabilities and care needs than before (Aaltonen et al., 2023). As our findings indicate, home care work has become more demanding, yet has to be done with limited resources. This inevitably has negative impacts on not only care workers wellbeing but also the quality of eldercare services (Van Aerschot et al., 2022).

NPM creates new values and ignores long-standing values such as human dignity, justice and individuality, which are the basis of the traditional public service ethos, the uniqueness of the individual, welfare, and social justice (Diefenbach, 2009). These traditional values of the public sector are important for care workers and these values have been broken when applying the ideologies of NPM to care work. Care work is relational work, where knowledge of the client and autonomy make it possible to adapt care individually, which is crucial for conducting satisfactory work (Kamp, 2012). Moreover, it is important that care workers are able to act according to their values and maintain “moral agency” to prevent and reduce ethical burden (Paavolainen et al., 2023).

In the public sector, the NPM reform has primarily been applied due to cost-saving purposes with little focus on its other opportunities, such as customer orientation and effectiveness of services. From the care workers’ perspective, the NPM oriented standardization and monitoring of work tasks has restricted ability to use professional skills, knowledge, and autonomy, which may lead to distrust towards supervisors among employees (Strandås et al., 2019). In eldercare services, management has changed from a position in a professional hierarchy to a position in a managerial hierarchy (Rasmussen, 2012). Management level actions tend to be experienced as one-sided expectations for care workers to perform tasks without the organization having any obligations to take care of the employees' wellbeing. In the same way that clients are defined as numerical results, care workers also become faceless performers whose individual and personal characteristics and needs are ignored.

Our study has some limitations. Data was collected via social media platforms targeted for care workers who have decided to leave their jobs. We acknowledge that these participants' experiences may be more negative compared to workers’ who have stayed in their jobs. However, we find it highly important that light is shed also to the most challenging experiences leading to decisions—not only intentions—to leave the workforce. The online data collection tool allowed participants to express their experiences openly, but it of course excluded participants who do not use social media or were unable/unwilling to share their experiences in written form. Another limitation is the absence of information on respondents' gender and the time when they had left their jobs. Like most qualitative studies, we didn’t aim for data objectivity and generalization (Symon & Cassell, 2012) but sought a deep understanding of participants' experiences through individual and collective interpretations during data analysis.

As a conclusion, NPM-driven structural changes in the eldercare sector have led to a lack of dialog and joint goals between management and care workers. Consequently, care workers experience inadequacy and helplessness at work, as well as a constant ethical conflict between their own professional obligations and the requirements set by the management level. Care workers' reasons for leaving the field do not reflect only organizational solutions (e.g., Selander et al., 2022) but also national and global reforms impacting care workers working conditions. It seems that a well-intentioned NPM ideology has turned against itself reducing the wellbeing of workers and the quality of eldercare services. NPM contributes to the fragmentation of employees' and clients' needs into narrowly defined work tasks, leading to the loss of holistic understanding of care. This creates a negative cycle of care workers leaving the sector and older clients being at risk of inadequate support in their daily lives. As the population ages and care needs increase worldwide, it is highly important to shift this course by paying more attention to the organization and resourcing of home care services from the perspective of care workers’ and older clients’ wellbeing and care needs. This requires acknowledgment and involvement of care workers expertise and knowledge in the development of care services and aging policies.

## Data Availability

The data is currently owned and managed by Marjo Ring. It will be openly available in 2025 at The Finnish Social Science Data Archive: https://www.fsd.tuni.fi/fi/.
